# Supplementary material for: A small viral potassium ion channel with an inherent inward rectification
Source: Channels (Austin). 2019 Apr 22;13(1):124–35. doi: 10.1080/19336950.2019.1605813 (PMC6527081; doi:10.1080/19336950.2019.1605813)
Supplement: Supplemental Material [file kchl-13-01-1605813-s001.pdf]

Supplement

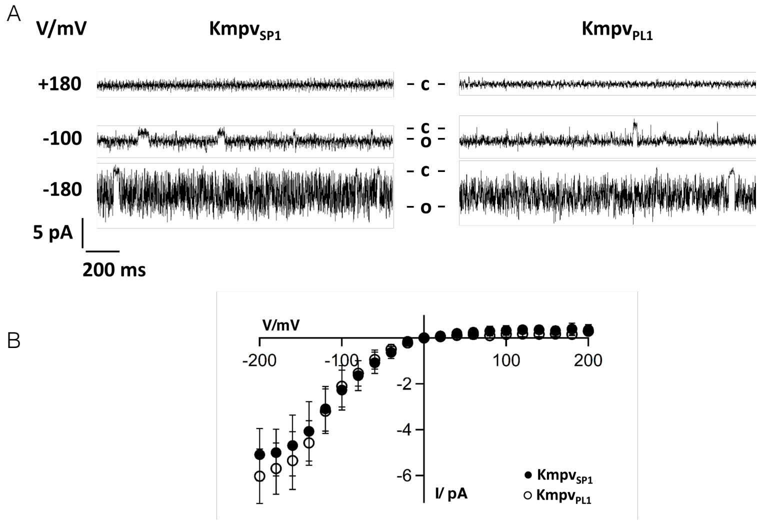

Figure 1 supplement: Kmpv$_{PL1}$ has the same inward rectification as Kmp$_{SP1}$. (A) Exemplar current traces of Kmpv$_{PL1}$ and Kmpv$_{SP1}$ activity over a range of clamp voltages in symmetrical KCl buffer (100 mM KCl + 10 mM HEPES, pH 7). Both channel proteins were translated *in vitro* as in Fig. 4. The Kmpv$_{PL1}$ channel exhibits the same high open probability with flicker type fluctuations as Kmpv$_{SP1}$. **(B)** Mean I/V relations of average currents (mean ± sd; n≥9) of both channels measured over clamp steps of 60 sec.

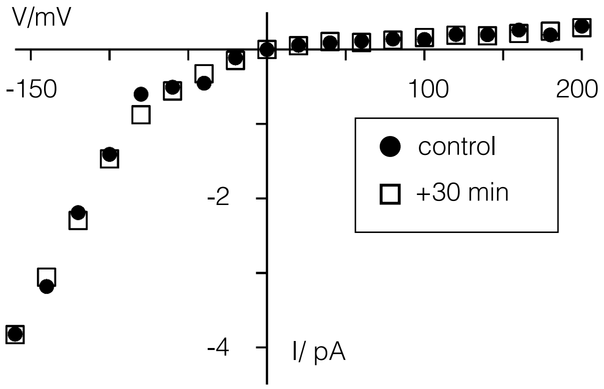

Figure 2 supplement: Long term incubation of Kmpv$_{SP1}$ in pure KCl buffer does not compromise rectification. I/V relation of mean currents measured in planar lipid bilayer with Kmpv$_{SP1}$ protein immediately after functional reconstitution (circles) and 30 min later (squares). Data were recorded as in Fig. 4.

```
KmpvSP1 ------------------------------------------------------------
Kir1.1  MNASSRNVFDTLIRVLTESMFKHLRKWVVTRFFGHSRQ--------RARLVSKDGRCNIE
Kir6.1  MLARKSIIPEEYVLARIAAENLRKP-----------RIRDRLP---KARFIAKSGACNLA
Kir2.1  MGSVRT---NRYSIVSSEEDGMKLATMAVANGFGNGKSKVRTRQQCRSRFVKKDGHCNVQ
Kir3.1  MSALRRKFGDDYQVVTTSSSGSGLQ----PQGPGQDPQQQLVPKKKRQRFVDKNGRCNVQ

KmpvSP1 ------------------MTPID---KFKLIVIV-----------ALLYGFIYSRMDPEE
Kir1.1  FGNVEAQS-RFIFFVDIWTTVLDLKWRYKMTIFITAFLGSWFFFGLLWYAVAYIHKDLPE
Kir6.1  HKNIREQG-RFL--QDIFTTLVDLKWRHTLVIFTMSFLCSWLLFAIMWWLVAFAHGDIYA
Kir2.1  FINVGEKGQRYL--ADIFTTCVDIRWRWMLVIFCLAFVLSWLFFGCVLWLIALLHGDLDA
Kir3.1  HGNLGSETSRYL--SDLFTTLVDLKWRWNLFIFILTYTVAWLFMASMWWVIAYTRGDLNK
                           * :*   .  : ::             : : .   . *

KmpvSP1 F------------------GFSSPLDPYYFSFTTMSSVGYGDS--SPKTDRAKLLVMTQQ
Kir1.1  F-------HPSANHTPCVENINGLTSAFLFSLETQVTIGYGFRCVTEQCATAIFLLIFQS
Kir6.1  YMEKSGMEKSGLESTVCVTNVRSFTSAFLFSIEVQVTIGFGGRMMTEECPLAITVLILQN
Kir2.1  S-------KEG---KACVSEVNSFTAAFLFSIETQTTIGYGFRCVTDECPIAVFMVVFQS
Kir3.1  A-------HVG-NYTPCVANVYNFPSAFLFFIETEATIGYGYRYITDKCPEGIILFLFQS
                           . .   .: * : .  ::*:*     : :   .   :.:*.

KmpvSP1 V-------FIGGEIL-KLLMFKRKSK
Kir1.1  ILGVIINSFMCGAILAKISRPKKRAKTITFSKNAVISKRGGKLCLLIRVANLRKSLLIGS
Kir6.1  IVGLIINAVMLGCIFMKTAQAHRRAETLIFSRHAVIAVRNGKLCFMFRVGDLRKSMIISA
Kir2.1  IVGCIIDAFIIGAVMAKMAKPKKRNETLVFSHNAVIAMRDGKLCLMWRVGNLRKSHLVEA
Kir3.1  ILGSIVDAFLIGCMFIKMSQPKKRAETLMFSEHAVISMRDGKLTLMFRVGNLRNSHMVSA
        :       .: * :: *    :..  * : : :: :..*           ..:: :  :.

KmpvSP1 -----------------------------------------------------------
Kir1.1  HIYGKLLKTTVTPEGETIILDQININFVVDAGNENLFFISPLTIYHVIDHNSPFFHMAAE
Kir6.1  SVRIQVVKKTTTPEGEVVPIHQLDIPVDNPIESNNIFLVAPLIICHVIDKRSPLYDISAT
Kir2.1  HVRAQLLKSRITSEGEYIPLDQIDINVGFDSGIDRIFLVSPITIVHEIDEDSPLYDLSKQ
Kir3.1  QIRCKLLKSRQTPEGEFLPLDQLELDVGFSTGADQLFLVSPLTICHVIDAKSPFYDLSQR

KmpvSP1 ------------------------------------------------------------
Kir1.1  TLLQQDFELVVFLDGTVESTSATCQVRTSYVPEEVLWGYRFAPIVSKTKEGKYRVDFHNF
Kir6.1  DLANQDLEVIVILEGVVETTGITTQARTSYIAEEIQWGHRFVSIVTE-EEGVYSVDYSKF
Kir2.1  DIDNADFEIVVILEGMVEATAMTTQCRSSYLANEILWGHRYEPVLFE-EKHYYKVDYSRF
Kir3.1  SMQTEQFEIVVILEGIVETTGMTCQARTSYTEDEVLWGHRFFPVISL-EEGFFKVDYSQF

KmpvSP1 ------------------------------------------------------------
Kir1.1  SKTVEVETPH--------------------------------------------------
Kir6.1  GNTVKVAAPR--------------------------------------------------
Kir2.1  HKTYEVPNTP--------------------------------------------------
Kir3.1  HATFEVPTPPYSVKEQEEMLLMSSPLIAPAITNSKERHNSVECLDGLDDITTKLPSKLQK

KmpvSP1 ------------------------------------------------------------
Kir1.1  ------------C-----------------------AMCLYNEKDVRARMKRGYDNP---
Kir6.1  ------------CSARELDEKPSILIQTLQKSELSHQNSLRKRNSMRRNNSMRRNNSIRR
Kir2.1  -----------LCSARDLAEKKYILSN-------ANSFCYENEVALTSKEEDDSENGVPE
Kir3.1  ITGREDFPKKLLRMSSTTSEKAYSLGDLPMKLQRISSVPGNSEEKLVSKTTKMLSDPMSQ

KmpvSP1 -------------------------------------
Kir1.1  ----------------------NFILSEVNETDDTKM
Kir6.1  NNSSLMVPKVQ-------------FMTPEGNQNTSES
Kir2.1  STSTDTPPDID------LHNQASVPLEPRPLRRESEI
Kir3.1  SVA-DLPPKLQKMAGGAARMEGNLPAKLRKMNSDRFT
```

Figure 3 supplement: Viral inward rectifier shares no structural similarity with canonical Kir channels. Alignment of $Kmpv_{SP1}$ with canonical Kir channels. The four major functional subgroups (Hibino et al., 2010) of Kir channels are represented by the human Kir1.1 (BAG36779.1), Kir2.1 (NP_000211), Kir3.1 (P48549) and Kir6.1 (Q15842.1). Alignment performed with MUSCLE algorithm (https://www.ebi.ac.uk/Tools/msa/muscle/). Kir typical AAs are highlighted in the Kir2.1 sequence including: i) two Cys residues and charged AAs in the filter (in blue; Baronas and Kurata 2014), ii) the critical AA, which determines between weak and strong rectification (in green; Hibero et al., 2010; Baronas and Kurata, 2014), iii) residues which induce strong rectification when an acidic substitution is introduced in Kir6.2 (in turquoise; Baronas and Kurata, 2014; Nichols and Lee, 2018), (iv) AAs for PIP_2 interaction (in red; Hibero et al. 2010), (v) charged AAs on the wall of the cytoplasmic pore (in yellow; Fujiwara and Kubo 2006). Asterisks (*) indicate conserved residues, colons (:) indicate residues with strongly similar properties, and periods (.) indicate residues with weakly similar properties.
